# Two Novel Pathogenic Variants Confirm *RMND1* Causative Role in Perrault Syndrome with Renal Involvement

**DOI:** 10.3390/genes11091060

**Published:** 2020-09-08

**Authors:** Dominika Oziębło, Joanna Pazik, Iwona Stępniak, Henryk Skarżyński, Monika Ołdak

**Affiliations:** 1 Department of Genetics, Institute of Physiology and Pathology of Hearing, 02-042 Warsaw, Poland; d.ozieblo@ifps.org.pl (D.O.); neurogenetyka@protonmail.com (I.S.); 2 Postgraduate School of Molecular Medicine, Medical University of Warsaw, 02-091 Warsaw, Poland; 3 Department of Transplantation Medicine, Nephrology and Internal Diseases, Medical University of Warsaw, 02-091 Warsaw, Poland; jt.pazik@gmail.com; 4 Oto-Rhino-Laryngology Surgery Clinic, Institute of Physiology and Pathology of Hearing, 02-042 Warsaw, Poland; h.skarzynski@ifps.org.pl

**Keywords:** *RMND1* (required for meiotic nuclear division 1 homolog), Perrault syndrome, renal disease, hearing loss, ovarian dysfunction, COXPD11 (combined oxidative phosphorylation deficiency), mitochondria

## Abstract

*RMND1* (required for meiotic nuclear division 1 homolog) pathogenic variants are known to cause combined oxidative phosphorylation deficiency (COXPD11), a severe multisystem disorder. In one patient, a homozygous *RMND1* pathogenic variant, with an established role in COXPD11, was associated with a Perrault-like syndrome. We performed a thorough clinical investigation and applied a targeted multigene hearing loss panel to reveal the cause of hearing loss, ovarian dysfunction (two cardinal features of Perrault syndrome) and chronic kidney disease in two adult female siblings. Two compound heterozygous missense variants, c.583G>A (p.Gly195Arg) and c.818A>C (p.Tyr273Ser), not previously associated with disease, were identified in *RMND1* in both patients, and their segregation with disease was confirmed in family members. The patients have no neurological or intellectual impairment, and nephrological evaluation predicts a benign course of kidney disease. Our study presents the mildest, so far reported, *RMND1*-related phenotype and delivers the first independent confirmation that *RMND1* is causally involved in the development of Perrault syndrome with renal involvement. This highlights the importance of including *RMND1* to the list of Perrault syndrome causative factors and provides new insight into the clinical manifestation of *RMND1* deficiency.

## 1. Introduction

*RMND1* (required for meiotic nuclear division 1 homolog) is a nuclear gene that encodes a protein needed for proper functioning of mitochondria. Although the data on its exact role are still limited, it has been shown that the RMND1 protein belongs to a large mitochondrial inner membrane complex that supports translation of the mtDNA-encoded polypeptides [1,2], all of which represent essential structural components of the oxidative phosphorylation (OXPHOS) complexes. It has been proposed that RMND1 tethers mitochondrial ribosomes close to the sites where the primary mRNAs are matured, spatially coupling mitochondrial transcription with translation [3]. In line with this, *RMND1* pathogenic variants cause a generalized mitochondrial translation defect and are detected in patients with combined oxidative phosphorylation deficiency (COXPD11; MIM #614922), a severe recessive condition characterized by the presence of lactic acidosis, deafness, renal and liver dysfunction, central nervous system and muscle involvement with an onset at birth or early infancy [4,5].

In 2018, a different clinical presentation consistent with a diagnosis of Perrault syndrome (PRLTS) was associated with a known *RMND1* (c.713A>G; p.Asn238Ser) homozygous variant. The individual reported by Demain et al. suffered from sensorineural hearing loss (HL) and primary ovarian insufficiency (POI), defining clinical features of PRLTS, in addition to renal dysfunction and short stature. The phenotype was delineated based on exome sequencing data from a single patient [6]. Considering the absence of another reported disease-causing variant, a doubt may arise as to whether *RMND1* is related to PRLTS development. Here, we present novel data delivering the first independent confirmation that *RMND1* is causally involved in the development of PRLTS with chronic kidney disease.

## 2. Materials and Methods

### 2.1. Study Subjects

**Figure 1:**
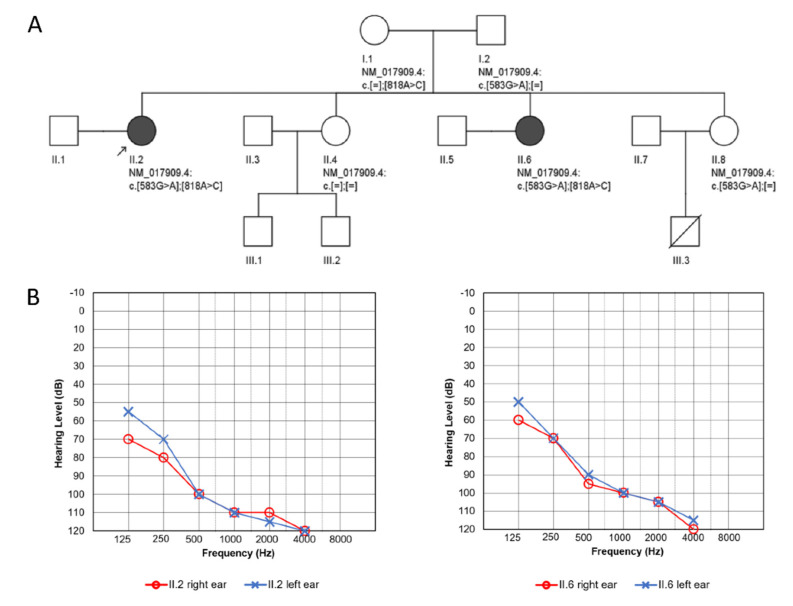
Pedigree and audiological data of the investigated family. (**A**) Pedigree showing affected family members (proband II.2, proband’s sister II.6) and the identified *RMND1* variants. (**B**) Pure tone audiometry results of the proband (left panel) and her sister (right panel) at the age of 32.

Two affected sisters from a nonconsanguineous Polish family, together with their parents and two other unaffected sisters, participated in the study (Figure 1A). The proband was born at term after an uneventful pregnancy, and her development in the first years of life was considered normal until the age of four, when bilateral HL was diagnosed. She received hearing aids at the age of six; the degree of HL progressed gradually and was accompanied by tinnitus and vertigo from the age of 31. No ear malformations were observed on temporal bone CT scans. Cochlear implantation was performed for the right ear at the age of 34 and for the left ear at the age of 36 with a good outcome. From the age of 17, she was under gynecological care due to irregular and scanty menstruation (menarche at age 14). Hypergonadotropic hypogonadism and small ovaries and uterus were recognized. Infertility was diagnosed, and hormone replacement therapy was introduced at the age of 28. Hypertension was diagnosed at the age of 31. At the age of 33, her left adrenal gland was removed because of lymphangioma, and chronic kidney disease (CKD) was diagnosed.

The proband’s younger sister was also born at term without any complications, and her development was normal. Bilateral HL was diagnosed at the age of 3. She does not have tinnitus or vertigo, and her HL is stable. She is using hearing aids sporadically and prefers to communicate with sign language. Evaluation for primary amenorrhea and delayed pubertal development at the age of 18 revealed gonadal dysgenesis with a normal female karyotype 46,XX. Vitamin B12 deficiency anemia and osteoporosis were diagnosed at the age of 26. At the age of 32, hypertension and CKD were recognized. Due to hypertension, enarenal and indapamide were implemented in the proband and amlodipine and torasemide in her sister. Both receive oestradiol and dydrogesterone supplementation to reduce the complications of POI.

Written informed consent was obtained from all participants. The study was approved by the ethics committee at the Institute of Physiology and Pathology of Hearing (KB.IFPS.25/2017) and performed according to the Declaration of Helsinki.

### 2.2. Nephrological and Neurological Examinations

The proband and her sister underwent thorough nephrological evaluation including whole blood count, electrolytes, venous blood gases, serum creatinine, calcium/phosphate balance, uric acid, lipid profile, urinalysis as well as urine albumin to creatinine ratio (UACR), urinary tract ultrasound with kidney size, and cortical thickness evaluation. To estimate glomerular filtration rate (eGFR), CKD-EPI (CKD Epidemiology Collaboration) creatinine equation was used [7]. A detailed neurological examination was performed. To assess the occurrence of neurological signs, the scale for assessment rating of ataxia—5th version (SARA) and Inventory of Non-Ataxia symptoms—6th version (INAS) were used [8,9].

### 2.3. Targeted HL Gene Panel, Data Analysis and Interpretation

Genomic DNA was extracted using a standard salting out procedure. Libraries were prepared with a custom HL 237-gene panel (SeqCap EZ Choice, Roche, Switzerland), containing genes related to PRLTS, i.e., *HSD17B4*, *HARS2*, *LARS2*, *TWNK*, *ERAL1*, *CLPP*, and *RMND1* and sequenced on a MiSeq Illumina platform. The quality control of raw FASTQ reads was performed, followed by adapter trimming and low quality reads removal with Trimmomatic [10]. Burrows–Wheeler Aligner [11] was used to map reads on hg38, followed by sorting and duplication removal using Samblaster [12]. Variant identification was done using multiple algorithms: HaplotypeCaller from GATK (Genome Analysis Toolkit) [13], Freebayes [14], DeepVariant [15], and MuTect2 [16]. Identified variants were annotated using Ensembl VEP [17] as well as multiple databases, including dbSNP [18], dbNSFP [18], GnomAD [19], ClinVar [20], and HGMD [21]. Inhouse databases of previously identified variants were used for annotation, to identify sequencing artifacts as well as variants common in the Polish population. The pathogenicity of identified variants was predicted based on the biochemical properties of the codon change and degree of evolutionary conservation using PolyPhen-2 [22], SIFT [23], Mutation Taster [24], LRT [25], and CADD [26]. Pathogenicity of the identified single nucleotide (SNV) and INDEL variants was evaluated by analyzing allele frequency, in silico predictions, annotations from public variant databases, matches in the inhouse variants database, and related medical literature. Evolutionary conservation was evaluated using GERP++ score [27]. Multiple protein sequence alignment was performed using COBALT [28], and variant localization across evolutionary diverse species was visualized with Jalview v2.11.1.0 software [29]. Detected variants were assigned according to standards and guidelines for the interpretation of sequence variants [30,31]. Selected probably causative variants were confirmed using direct Sanger sequencing and reported based on the *RMND1* NM_017909.4 and NP_060379.2 reference sequences.

## 3. Results

### 3.1. Clinical Presentation

The major clinical features of the proband, a 44-year old female, and her sister, a 36-year old female, were severe-to-profound bilateral sensorineural HL (Figure 1B) and ovarian dysfunction accompanied by CKD that developed in the fourth decade of life. Both had a normal stature. Laboratory findings on renal involvement, blood lactate concentration and core parameters of venous acid-base balance are given in Table 1, as shown by eGFR and UACR both patients were in stage G3, A1 of CKD [32]. The proband’s calculated one-year eGFR decline was −0.45 mL/min and in her sister −0.66 mL/min. On repeated ultrasound evaluations, the size and cortex thickness of the kidneys was slightly diminished, but generally, the kidneys’ dimensions did not change within a twelve-year follow up. The proband’s sister had a more complex nephrological profile. Although on ultrasound, both kidneys and their cortex were of normal size, on scintigraphy at the age of 32, substantial asymmetry of ERPL (effective renal plasma flow; 64% left, 36% right kidney) with uneven radiotracer accumulation in the right organ was found. It was interpreted as post-inflammatory scars even though the patient denied urinary tract infections.

Neurological assessment did not reveal any features of cerebellar, pyramidal or extrapyramidal syndromes either in the proband or her affected sister. They presented normal muscle tone and strength as well as reflexes in the upper and lower limbs. Both have completed higher education.

### 3.2. Identification of Pathogenic Variants

**Figure 2:**
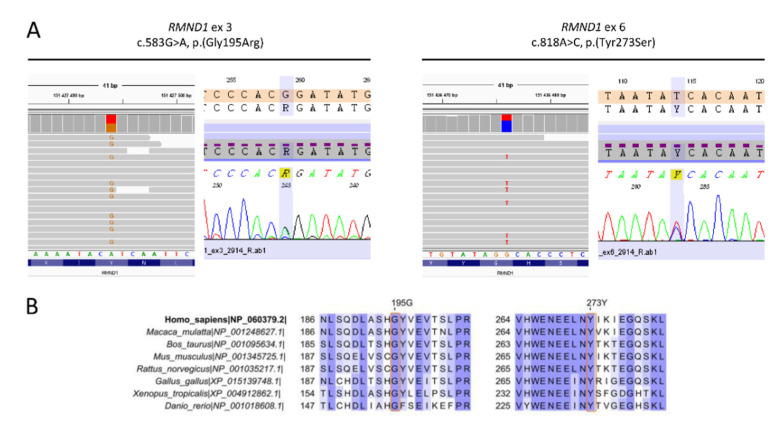
Genetic data of the investigated family. (**A**) Results of next-generation sequencing (NGS) and Sanger sequencing showing c.583G>A transition (p.Gly195Arg) and c.818A>C transversion (p.Tyr273Ser) in the *RMND1* gene. (**B**) Multiple protein sequence alignment of selected RMND1 regions among different species.

After performing next-generation sequencing (NGS), two heterozygous variants, c.583G>A and c.818A>C in *RMND1*, corresponding to missense changes p.Gly195Arg and p.Tyr273Ser, respectively, were identified in the proband (Figure 2A). The vast majority of computational algorithms predicted a probably pathogenic character of detected variants, and they were identified only in heterozygous, individual cases in the gnomAD population database (Table 2). Conservation analyses showed 100% identity of the analyzed regions among all tested species (Figure 2B), with GERP++ scores of 4.57 and 5.95. Based on the applicable standards and guidelines, we have classified the identified *RMND1* variants as likely pathogenic. No other pathogenic variants related to isolated or syndromic hereditary HL, in particular to PRLTS, were found. The same *RMND1* variant constellation was identified in her affected younger sister. Both parents and another healthy sister were heterozygous carriers of one of the *RMND1* variants. In the third sister, none of the *RMND1* variants was identified (Figure 1A).

## 4. Discussion

Our clinical and genetic investigation shows that a combination of HL, ovarian dysfunction, and CKD constitutes a milder end of the *RMND1*-related phenotypic spectrum. Presence of the three clinical features can be defined as Perrault-like syndrome [6], PRLTS with renal involvement or just PRLTS with a respective consecutive number, according to the nomenclature used by OMIM (Online Mendelian Inheritance in Man, https://omim.org/), where subsequent numbers are assigned to a syndrome in order to distinguish the causative gene. PRLTS is characterized by the presence of sensorineural HL in both males and females and ovarian dysfunction ranging from gonadal dysgenesis to POI in females. These are the two PRLTS cardinal features; however, in some individuals additional, usually neurological conditions (e.g., developmental delay, cognitive impairment, ataxia or sensory axonal neuropathy) have been also reported (Table 3) [33]. Taking into account the heterogeneity of PRLTS phenotypic manifestations, in our opinion, it seems justified to recognize *RMND1* as the seventh PRLTS gene, where renal involvement represents an additional characteristic finding and no neurological signs or symptoms are found (neither in the patient reported by Demain [6] nor in our patients).

Kidney function is frequently affected in patients with RMND1 deficiency. Analyzing a large group of patients with COXPD11 due to *RMND1* pathogenic variants, Ng et al. found that renal involvement was present in more than two thirds of patients [4]. It was manifested by cystic dysplasia, renal tubular acidosis (persistent hyponatremia and hyperkalemia), end stage renal failure with subsequent kidney transplantation, anemia, proteinuria or CKD at different stages. The single, so far described, patient with PRLTS and *RMND1* homozygous pathogenic variant [6] had distal renal tubular acidosis with hyperchloremic metabolic acidosis, a normal anion gap, mildly elevated uric acid, low urine citrate levels, normal calcium levels, and a normal renal ultrasound. CKD was mentioned, but exact kidney function has not been given. In our patients, we found CKD of mild to moderate severity. The proband was affected by metabolic acidosis with normal fasting lactic acid concentration, hyperkalemia, normal chloride, and a normal anion gap. In her sister, we did not find metabolic acidosis, although the fasting lactic acid concentration was slightly above normal values. Both sisters presented with hypertension that may be secondary to CKD, and applied antihypertensive medications might have had an influence on electrolyte abnormalities. The calculated yearly filtration losses that we assessed in the patients were similar to the value of eGFR slope (−0.48 mL/min) found in women aged 35 to 49 years and renal stage IIIa (45–59 mL/min) [34]. This, together with a low-grade UACR of our patients, predicts a benign kidney disease course and makes reaching kidney failure and a requirement of renal replacement therapy less likely [32].

Ovarian dysfunction (ovarian atrophy and hypergonadotropic hypogonadism) as a consequence of *RMND1* pathogenic variants has been previously reported only once in a patient described by Demain et al. [6] and in none of the approximately 40 patients with OXPHOS deficiency. This could be explained by the early, prepubertal age at which the majority of children were investigated [4,5,35,36]. In only two patients examined at the age of 14 and 17, no reference was made to their sexual development [36]. Thus, at the moment, it is not clear how frequently ovaries are affected in patients with RMND1 deficiency.

RMND1 is a nuclear-encoded protein involved in mitochondrial translation. Disruption of this process is a well-known mechanism leading to PRLTS development (Table 3). In this study, we have identified two likely pathogenic *RMND1* variants not previously associated with disease. Presence of the detected *RMND1* variants in a trans configuration is consistent with the autosomal recessive mode of inheritance. Our study provides an independent confirmation on the causative role of *RMND1* in Perrault syndrome with renal involvement. Hearing loss and renal dysfunction are typical for of *RMND1*-related disorders. These two clinical features accompanied by ovarian dysfunction were present in our patients and they are consistent with the phenotype reported in the original study by Demain et al. [6]. The identification of two ultra-rare *RMND1* variants that are in a *trans* configuration, co-occur in two affected family members (having an almost identical phenotype) and do not co-occur in two other healthy siblings, strongly supports their pathogenic potential.

**Figure 3:**
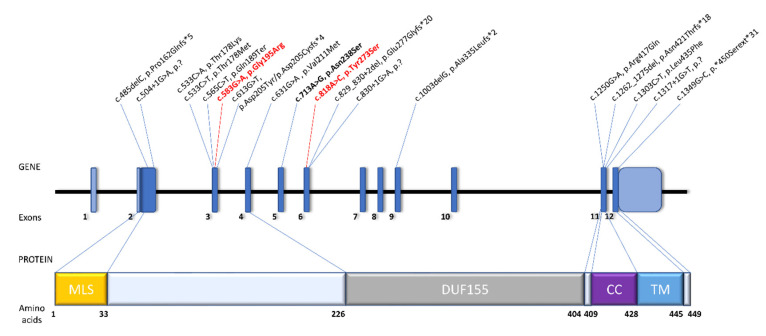
Schematic representation of *RMND1* gene and protein organization. Gene and protein structure is depicted based on the canonical transcript NM_017909.4 and reference protein sequence NP_060379.2. Previously reported *RMND1* pathogenic variants involved in development of combined oxidative phosphorylation deficiency (COXPD11) are written in black. Variants identified in this study are shown in red. Bolded are variants involved in the development of Perrault syndrome (PRLTS) with renal involvement. Abbreviations: MLS, mitochondrial localization sequence; DUF155, domain of unknown function; CC, coiled-coil; TM, transmembrane.

One of the identified variants (p.Gly195Arg) localizes close to the DUF155 domain at the protein N-terminus and the second one (p.Tyr273Ser) within the DUF155 domain (Figure 3). Considering that *RMND1* has three protein-encoding transcripts all of which contain the DUF155 domain [1], one may assume that all three proteins arising from the p.(Tyr273Ser)-carrying allele will be dysfunctional. It is not applicable for the second *RMND1* variant, that will affect two out of three alternative transcripts, leaving some functional RMND1 protein in the cells. Although the tissue-specific ratio of *RMND1* transcripts remains unknown, this observation may provide a possible explanation for the milder phenotype in our patients. It could also be owed to some other yet unidentified modifying factors. It is still a conundrum why the single patient with a homozygous p.(Asn238Ser) variant, localizing within the DUF155 domain, presented a relatively mild phenotype resembling PRLTS [6], in contrast to the, currently, four other patients with the same causative variant and a more severe infantile-onset multisystem disorder [4,5,37].

## 5. Conclusions

In summary, we report two novel *RMND1* likely pathogenic variants leading to the mildest, so far reported, *RMND1*-related phenotype that corresponds to PRLTS with renal involvement. It was identified in two adult siblings with a very similar clinical presentation. Our study highlights the importance of including *RMND1* to the list of PRLTS causative factors and directs attention to ovaries as yet another organ affected by RMND1 deficiency. Future functional studies could be helpful to clarify the molecular mechanisms underlying the differences in phenotype severity of *RMND1*-related disorders.

## Figures and Tables

**Table 1:** Laboratory results of the proband and her sister.

	RBC (T/L)	Hb (g/dL)	Creatinine (mg/dL)	eGFR CKD EPI (mL/min)	Acid Base Venous Balance	Blood Lipids (mg/dL)	Calcium (mg/dL)	Phosphates (mg/dL)	PTH (pg/mL)	UACR (mg/g)
**proband**	4.49 (4.2–6.3)	13.3 (12–16)	**1.53** (0.6–1.3)	**41** (>90)	pH **7.31** (7.35–7.45) HCO_3_^−^ **20.7** (22–26 mmol/L) BE −1.7 (−2 to +2mmol/l) Anion gap 12 (12 ± 4 mEq/L) Cl^−^ 105 (98–106 mmol/L) Lactic acid 1.2 (0.5–1.6 mmol/L) **K^+^ 5.4** (3.4–4.5 mEq/L) Na^+^ 137 (136–146 mEq/L)	T chol 159 (<190) HDL 71 (35–65) LDL 72 (<115) TG 76 (<150)	9.2 (8.5–10.1)	3.6 (2.5–4.9)	**72.6** (12–68.3)	2.9 (<30)
**proband’s** **sister**	4.08 (4.2–6.3)	12.4 (12–16)	**1.38** (0.6–1.3)	**49** (>90)	pH 7.35 (7.35–7.45) HCO_3_^−^ 22 (22–26 mmol/L) BE −0.6 (−2 to +2mmol/L) Anion gap 8.9 (12 ± 4 mEq/L) Cl^−^ 105 (98–106 mmol/L) Lactic acid **1.7** (0.5–1.6 mmol/L) **K^+^ 5.2** (3.4–4.5 mEq/L) Na^+^ 138 (136–146 mEq/L)	T chol **213** (<190) HDL 68 (35–65)LDL **145** (<115) TG 57 (<150)	9.4 (8.5–10.1)	3.6 (2.5–4.9)	**148.8** (12–68.3)	6.5 (<30)

Abnormal values are given in bold; reference values are in parentheses.

**Table 2:** Characteristics of *RMND1* variants detected in this study.

Variant cDNA Level	Variant Protein Level	Reference SNP ID	Population Frequencies			Pathogenicity Predictions					
			gnomAD	1000 Genomes	ESP 6500	SIFT	PolyPhen-2	Mutation Taster	LRT	CADD	ACMG Classification *
c.583G>A	p.(Gly195Arg)	rs776083030	0.00002388 (6/251308)	0	0	D (0.011)	PD (0.997)	D (1)	D (0)	D (29.7)	LP (PM2, PP1_M, PP3, PP4)
c.818A>C	p.(Tyr273Ser)	rs766739125	0.00000399 (1/250612)	0	0	D (0)	PD (1)	D (0.99)	N (0.001742)	D (26.4)	LP (PM2, PP1_M, PP3, PP4)

Abbreviations: D, damaging; PD, probably damaging; N, neutral; * ACMG classification criteria legend: LP, likely pathogenic; PM, moderate pathogenicity evidence; PP, supporting pathogenicity evidence; _M, moderate.

**Table 3:** Genes causally involved in the development of PRLTS and PRLTS-like features.

Gene (Locus)	Protein	Subcellular Localization	Function	Additional Clinical Features *	Inheritance Mode	Ref.
*CLPP* (19p13.3)	caseinolytic mitochondrial matrix peptidase proteolytic subunit	mitochondrial	mitochondrial protein degradation (component of a proteolytic complex)	• neurologic (e.g., ataxia, polyneuropathy, epilepsy, learning and developmental delay, spastic diplegia) • microcephaly • growth retardation	AR	[33,38,39,40,41,42,43]
*ERAL1* (17q11.2)	Era like 12S mitochondrial rRNA chaperone 1	mitochondrial	mitochondrial protein translation (assembly of mitochondrial ribosomal subunit)	• not reported	AR	[44]
*GGPS1* (1q42.3)	geranylgeranyl diphosphate synthase 1	cytoplasmic	acts on peroxisomal products, part of mevalonate pathway	• neurologic (muscular dystrophy, myopathy)	AR	[33,45]
*HARS2* (5q31.3)	histidyl-tRNA synthetase 2	mitochondrial	mitochondrial protein translation (synthesis of histidyl-transfer RNA)	• not reported	AR	[39,46,47]
*HSD17B4* (17q21.2)	hydroxysteroid 17-β dehydrogenase 4	peroxisomal	β-oxidation pathway for fatty acids in peroxisomes	• neurologic (e.g., ataxia, polyneuropathy, cerebellar atrophy, spastic diplegia, hypertonia, dysarthria, nystagmus, oculomotor apraxia, tremor, delayed motor development, cognitive impairment) • growth retardation • skeletal (e.g., pes cavus, pes equinovarus, scoliosis)	AR	[39,41,48,49,50,51]
*LARS2* (3p21.31)	leucyl-tRNA synthetase	mitochondrial	mitochondrial protein translation (synthesis of leucyl-transfer RNA)	• neurologic (e.g., ataxia, cerebellar syndrome, epilepsy, developmental delay, intellectual impairment, behavior disorder, leukodystrophy) • macrocephaly	AR	[33,39,41,52,53,54,55,56,57,58,59,60]
*PEX6* (6p21.1)	peroxisomal biogenesis factor 6	peroxisomal	peroxisomal protein import (ATPase activity)	• not reported	AR	[33]
*RMND1* (6q25.1)	required for meiotic nuclear division 1 homolog	mitochondrial	mitochondrial protein translation	• kidney disease • short stature	AR	[6] Present study
*TFAM* (10q21.1)	transcription factor A, mitochondrial	mitochondrial	key mitochondrial transcription factor	• intellectual impairment	AR	[33]
*TWNK* (10q24.31)	twinkle mtDNA helicase	mitochondrial	mitochondrial DNA replication and transcription (unwinds double-stranded DNA)	• neurologic (e.g., ataxia, polyneuropathy, limb paresis, muscle atrophy, muscle weakness, atrophy of cerebellum, diminished cervical enlargement, epilepsy, impaired eye movements, nystagmus, dysarthria)	AR	[39,41,61,62,63,64,65]

* Clinical features additional to hearing loss (HL) and ovarian dysfunction observed in some patients. AR, autosomal recessive.
